# Complete Genome Sequences of Three Staphylococcus pseudintermedius Strains Isolated from Botswana

**DOI:** 10.1128/genomeA.01599-17

**Published:** 2018-03-08

**Authors:** Mohamed A. Abouelkhair, Riley Thompson, Matthew C. Riley, David A. Bemis, Stephen A. Kania

**Affiliations:** a Department of Biomedical and Diagnostic Sciences, University of Tennessee College of Veterinary Medicine, Knoxville, Tennessee, USA; b Center for Genome Sciences, United States Army Medical Research Institute of Infectious Diseases, Fort Detrick, Maryland, USA

## Abstract

We report here the first whole-genome sequences for 3 strains of Staphylococcus pseudintermedius (112N, 113N, and 114N) isolated in Africa. Samples of this opportunistic pathogen were collected from nasal swabs obtained from healthy carrier dogs in Botswana. The sequence information will facilitate spatial phylogenetic comparisons of staphylococcal species and other bacteria at the genome level.

## GENOME ANNOUNCEMENT

Staphylococcus pseudintermedius is a Gram-positive, coagulase-positive bacterium commonly found as part of the normal skin and nasal flora of healthy dogs (1, 2). It is frequently associated with skin, soft tissue, and wound infections and less often with pneumonia and urinary tract infections similar to those caused by Staphylococcus aureus (2, 3). S. pseudintermedius infrequently colonizes the nasal mucosa of humans, where it has been associated with infections primarily from bite wounds or in immunocompromised individuals (3).

Most phenotypic and genotypic studies have been performed with methicillin-resistant S. pseudintermedius strains (1, 4–6). However, variability of phenotypic and genotypic features, including virulence, molecular epidemiology, and biological characteristics, have not been fully explored in methicillin-susceptible S. pseudintermedius (MSSP) isolates (7).

Whole-genome shotgun projects for S. pseudintermedius strains have been deposited at DDBJ/ENA/GenBank, including European S. pseudintermedius isolates (4, 6) and genomes from dominant clonal lineages in North America (6), but no complete genomes for S. pseudintermedius strains from the African continent are available and distinct geographical strain differences have been observed in this species (1, 4, 6, 8–13).

Whole-genome sequencing of MSSP isolates from Botswana will facilitate identification of genetic relatedness of these isolates with previously published MRSP and MSSP strains (1, 4–6, 12, 13) and facilitate evolutionary studies of staphylococcal species and other bacteria at the genome level.

All Botswana S. pseudintermedius strains were sequenced using Illumina MiSeq (Illumina, Inc., USA), with two runs (75 bp forward and reverse) generating a total of 1,091,288, 1,219,855, and 1,171,515 reads for S. pseudintermedius 112N, 113N, and 114N strains, respectively, with overall coverage of >250-fold. The resulting reads for each sequence were assembled using Geneious version 11.0.4 (14). The NCBI Prokaryote Genome Annotation Pipeline software version 4.3 was used for annotating the genomes.

The 112N genome is 2,516,677 bp (291 contigs of 500 bp or greater) with a 37.7% GC content, 2,375 predicted coding sequences, and 67 predicted RNAs. The 113N genome is 2,490,161 bp (96 contigs) with a 37.6% GC content, 2,257 predicted coding sequences, and 72 predicted RNAs. The 114N genome is 2,546,835 bp (84 contigs) with a 37.5% GC content, 2,301 predicted coding sequences, and 72 predicted RNAs. The strains have unique multilocus sequence types (ST) (12) including ST887, ST888, and ST889 for S. pseudintermedius 112N, 113N, and 114N, respectively.

### Accession number(s).

These whole-genome sequences of Staphylococcus pseudintermedius 112N, 113N, and 114N strains have been deposited at DDBJ/ENA/GenBank under the accession numbers PJUS00000000, PJUR00000000, and PJUQ00000000, respectively. The Staphylococcus pseudintermedius 112N, 113N, and 114N versions described in this paper are versions PJUS02000000, PJUR01000000, and PJUQ01000000, respectively.
